# Sirenomelia in a Cameroonian woman: a case report and review of the literature

**DOI:** 10.12688/f1000research.1-6.v2

**Published:** 2012-09-06

**Authors:** Frederick LI Morfaw, Philip N Nana

**Affiliations:** 1 Department of Obstetrics and Gynaecology, Faculty of Medicines and Biomedical Sciences, University of Yaoundé, Yaoundé, Cameroon

## Abstract

Sirenomelia is a rare congenital malformative disorder characterized by fusion of the lower limbs giving a characteristic mermaid-like appearance to the affected foetus. We report a case of sirenomelia occurring in a 19 year old Cameroonian woman following premature rupture of membranes and associated cord prolapse. This is the first documented case in this country. We highlight some of the cultural myths associated with this disorder and discuss our findings relative to the present literature and related controversies on its etiopathogenesis.

## Introduction

Sirenomelia is an extremely rare congenital malformative disorder, which is often fatal ^1^. Its incidence is estimated at about 1 in 100,000 pregnancies ^2^, and cases have been reported from all ethnic groups worldwide ^3^. This anomaly predominantly affects males (sex ratio 2.7:1) ^4^, and is frequent among one of two monozygotic twins ^5^. The most prominent yet inconstant feature of this malformative disorder is the complete or partial fusion of the lower limbs into a single lower limb ^3^. The resultant infant bears a resemblance to the mermaid of ancient Greek mythology ^6^. The disorder has equally been referred to as symmelia, sympodia monopodia, sympus, but most commonly as the ‘mermaid syndrome’ since the fusion of the lower limbs gives a characteristic mermaid-like appearance ^7^.

In the African context, such mermaid-like babies are referred to as ‘mammy-water babies’, and bear an evil connotation associated with witchcraft and sorcery.

The underlying visceral anomalies are usually such that the syndrome is incompatible with life ^3^, yet there are a number of reported cases of surviving infants with this condition in the English literature ^8– 12^.

We report here the first documented case of sirenomelia in Cameroon, and discuss our findings in relation to the present literature and related controversies of its etiopathogenesis.

## Case report

Miss N.N, 19 years old G2P0010, a single student at 38 weeks gestation according to her last menstrual period was referred to the Yaoundé Central Maternity for the management of cord prolapse at term. Her history revealed premature rupture of membranes 04 days prior to her consultation, with continuous per vaginal flow of clear liquor. She declares that foetal movements had been present prior to membrane rupture. She eventually developed uterine contractions 4 days later with an associated cord prolapse visible at the introitus. This pushed her to consult at a health centre from where she was referred to our hospital.

In her past medical history, she was not diabetic nor did she bear any known chronic pathologies. There was no family history of diabetes nor malformations. Her first pregnancy two years earlier ended up in a clandestine voluntary termination of pregnancy by endo-uterine aspiration in the first trimester without any recorded complications. This pregnancy resulted from a non-consanguineous union with a 23 year old student. This pregnancy was very poorly followed up in a local health centre with two antenatal consultations. The few tests that were carried out did not reveal any anomalies, but no ultrasound was done. The evolution of the pregnancy so far had been uneventful without any history of teratogenic drug intake or traditional concoctions.

**Figure 1.**
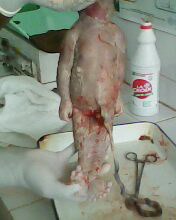
Complete morphological view showing distended abdomen, fused lower limbs and maceration around the neck.

On admission the patient was hemodynamically stable, afebrile, with a symphysis-fundal height of 26 cm, absent foetal heart tones, a foetus in cephalic presentation, and a non-pulsating third degree cord prolapse. The working diagnosis of a prolonged rupture of membranes complicated by third degree cord prolapse and intrauterine foetal death was made. The patient was induced using oxytocin infusions, and was placed on prophylactic parenteral antibiotics. Labour evolved normally and within 5 hours she expelled a dead first degree macerated foetus with the following morphological abnormalities: (see Figure 1– Figure 6).

**Figure 2.**
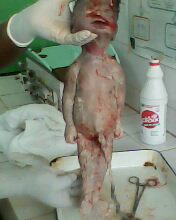
Complete morphological view of sirenomelic foetus.

**Figure 3.**
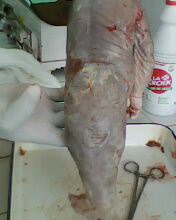
Posterior view showing fused lower limbs, imperforate anus.

**Figure 4.**
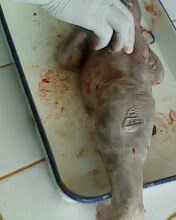
Imperforate anus.

**Figure 5.**
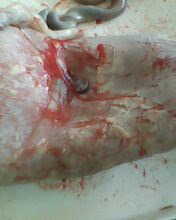
Photograph of the groin area showing the undetermined external genitalia, and absent urinary meatus.

**Figure 6.**
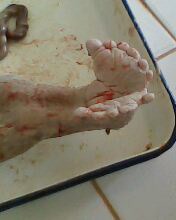
Photograph showing the fused feet with 11 toes.

- Distended abdomen
- umbilical cord with a single artery
- Undetermined sex (absent external genitalia with only a 2×3 cm tag marking the position of the genitalia).
- Imperforate anus
- absence of a urinary meatus
- fused lower segment of the body below the pelvis into a single lower limb, with two feet fused posteriorly giving a single flipper-like foot with eleven toes spread out in a fan-like pattern (Mermaid-like or ‘mammy-water’). The foot was oriented anteriorly relative to the trunk, and external palpation gave the impression of probably two femurs and two tibias.

There was complete placental delivery and uterine revision was done removing membranous debris.

The birth weight of 2.3Kg at 38 weeks gestation reflected intrauterine growth restriction.

Traditional and cultural beliefs precluded autopsy, and the corpse was handed over to the family for burial. The patient was maintained on parenteral antibiotics for 48 hours then oral relay was done on day 2 post-partum. She received adequate post-partum counselling and was discharged on that day.

## Discussion

Sirenomelia or mermaid syndrome is an abnormal development of the caudal region of the body involving varying degrees of fusion of the lower limbs with or without bony defects ^13^. It is usually associated with other visceral defects such as hypoplastic lungs, cardiac agenesis, absent genitalia, digestive defects, absent kidney and bladder, vertebral and central nervous system defects ^14, 15^. Death usually results from obstructive renal failure due to renal agenesis or dysgenesis, with survival depending on adequate kidney functioning and renal outflow ^16^.

In our patient however we could not ascertain the nature of the full foetal anomalies given that an autopsy was never performed, and no pre-birth imaging was available. Furthermore, the 3 ^rd^ degree cord prolapse was an obvious cause of fetal death. The history was suggestive of foetal movements in the few days prior to membrane rupture, hence this foetus could have been born alive.

## Etiology and risk factors

The etiology of this multisystemic human malformation is unknown ^3^ and no teratogens have been found in humans ^17^. Recent evidence from mice models suggests it can have a genetic basis, yet much still needs to be done to define the role of candidate genetic factors in humans ^3^. Certain risk factors however exist.

Maternal diabetes has been described as an important risk factor for caudal malformations in general ^3^. However, with only about 0.5–3.7% of sirenomelia cases occurring in diabetic mothers ^13, 18, 19^, the association between maternal diabetes and sirenomelia has been described as weak ^20^. Our patient was not known to be diabetic.

The syndrome is also reported to be associated with twins, with about 15% to 20% of cases being derived from products of twin pregnancies ^5, 13^, most of them monozygotic ^5^. Reports actually indicate a 100–150 times higher incidence in monozygotic twins relative to dizygotic twins or singletons ^20^. In our patient, this was not a twin gestation, and there was no family history of twinning.

Also, exposure to heavy metals has been shown to be associated sirenomelia in humans ^22, 23^.

It is worth noting that although Lynch *et al.* ^24^ recognised an autosomal form of caudal dysgenesis, no chromosomal abnormalities are found in sirenomelia and it does not recur in families ^14^. This was a reassuring feature for our patient and should serve as a counseling feature for mothers bearing babies with this distressing anomaly.

## Ethiopathogenesis

The etiopathogenesis of this syndrome has been subject to a lot of debate over time. Numerous theories have been proposed to explain its origin.

Stevenson *et al.* ^21^ proposed the vascular steal theory. This theory suggests that there is shunting of blood via an abnormal abdominal artery arising from high up in the aorta towards the placenta. This leaves the caudal part of the embryo poorly perfused. Consequently there is hypoplasia of the vasculature distal to the artery leading to nutritional deficiency of the caudal half of the body ^23^. Hence there may be complete/incomplete agenesis of the caudal structures (kidneys, sacrum, and lower portions of the digestive tract) except the gonads which are intra-abdominal. There could also be vertebral dysgenesis, lower limb atrophy and inconstant lower limb fusion ^14^. The single umbilical artery in our patient favours this theory. However, Jaiyessimi *et al.* ^20^ reported a case of sirenomelia without this vitelline artery steal, indicating that factors other than vitelline artery steal could be responsible for sirenomelia in humans.

The defective blastogenesis theory regards sirenomelia as part of the caudal regression syndrome (CRS) ^3^, more recently referred to as caudal dysgenesis ^25, 26^. This is a rare congenital defect characterized by a broad spectrum of lumbosacral dysgenesis. According to the this theory, during the ultimate stages of gastrulation occurring by the third week of intra-uterine life, there is a defect in blastogenesis leading to a wide range of phenotypic manifestations on the caudal extremity ^25, 27^. Even though the syndrome was initially described by Duhamel ^28^ to include genitourinary and vertebral anomalies, phenotypic expression depends on the intensity, duration and initiation time of the underlying event ^3^. Some authors consider sirenomelia to be the most extreme form of this relentless condition ^3^.

However for authors such as Pinette *et al.* ^16^, the distinction between caudal regression syndrome and sirenomelia remains speculative.

A further theory described in the literature regards sirenomelia as part of the VACTERL syndrome. VACTERL syndrome involves vertebral, anal, cardiovascular, tracheal esophageal, renal and limb dysgenesis. There is a major overlap in the phenotypic manifestations of sirenomelia and VACTERL ^20^. In most cases, the distinction between sirenomelia sequence and VACTERL lies within the severity of the component defects, and the single lower limb in sirenomelia can be regarded as an indicator of other severe malformations, especially in the gastrointestinal and genito-urinary systems ^20^. In our patient the lack of an autopsy did not permit any assertions to be made as to the relationship with VACTERL.

Other theories exist, but given their controversies in the literature, they are not considered here. Yet the overlap in these syndromes/theories waters the debate in the scientific world as to the uniqueness or diversities of these syndromes.

## Classification

Stocker and Heifetz ^13^ classified the sirenomelia sequence into 7 types as shown be in Table 1.

**Table 1.** Classification of the Sirenomelic sequence adapted from Stocker and Heifetz ^13^.

Type	Characteristic
I	All thigh and leg bones are present
II	Single fibula
III	Absent fibula
IV	Partially fused femurs, fused fibulae
V	Partially fused femurs
VI	Single femur, single tibia
VII	Single femur, absent tibia

We did not have any radiographs and could therefore not classify our patient into any of these categories with certainty even though external palpation was in favour of a type I.

## Diagnosis

The past medical history of the patient could identify patients at risk. Antenatal diagnosis is possible on ultrasound and x-ray. Ultrasound is however the predominant diagnostic tool. Moreover, there is no open defect which could cause abnormal increases in alpha foeto-protein levels ^29^.

Ultrasound features permitting confirmation of the diagnosis include lack of tibia/fibula, a single femur, convergent femoral bones, bilateral renal agenesis, polycystic kidneys/renal agenesis, obstructive uropathy and intra-uterine growth retardation ^30^. The use of ultrasound in diagnosis is however not without difficulty. In sirenomelic foetuses, bilateral renal agenesis causes severe oligohydramnios thus limiting ultrasound evaluation of the limbs in the second and third trimesters ^31, 32^. However, in earlier gestational ages, the amniotic fluid volume may be sufficient to detect abnormal lower limbs. In such cases, we may notice in addition to abnormal lower limbs, bilateral renal dysgenesis, absent bladder, undetermined external genitalia, anorectal agenesis and lumbosacral agenesis ^4^. Other abnormalities may touch the cardiovascular system and abdominal walls.

Antenatal confirmation of the diagnosis justifies a therapeutic termination of the pregnancy.

At delivery, clinical evaluation is usually sufficient to confirm the diagnosis. In our case, the diagnosis was obvious given the fused lower limbs, single umbilical artery, and imperforate anus. However radiographic images are important if we are to be able to classify the condition according to the Stocker and Heifer classification ( Table 1) ^13^. An autopsy permits determination of the extent of the associated anomalies, but is usually of limited use in the African context where cultural norms and beliefs largely precludes its practice.

## Management and prognosis

Sirenomelia carries with it a very poor prognosis. Survival is largely dependent on the extent of visceral anomalies, especially obstructive renal failure due to renal agenesis/dysgenesis ^16^. In the case of antenatal diagnosis, a voluntary termination of pregnancy is advisable in order to avoid the physical and psychological stress to parents and the family. This decision however depends on the gestational age of the pregnancy, the severity of the malformations and of course the desires of the parents ^17^.

Recent reports indicate that about 50% of these infants are born alive after 8–9 months gestation ^17^. However most of them die within 5 days of life ^13^. The management of sirenomelia is difficult and expensive, and the outcome is unpredictable ^17^. The main therapeutic modality involves surgical and medical compensation aimed mainly at maintaining adequate renal function. Surgery to correct the anomaly and separate the fused limbs is usually not a priority as there is no guarantee of its success, and it carries with it an increased risk of compromising the life of an already delicate infant.

There have been reports of surviving sirenomelic foetuses ^8– 12^. Pertinent amongst these is the case of the surviving infant with sirenomelia associated with absent bladder reported by Stanton *et al.* ^12^. This infant underwent 5 surgeries before the age of 4 years and continues to be bedridden and dependent. In the case described by Pinette *et al.* ^16^, the infant received a renal transplant using a cadaveric donor kidney. By publication time, this I infant was 5 years old with normal renal and cognitive development for her age. However, separation of the lower limbs was indefinitely delayed due to concerns regarding disruption of blood supply to abdominal organs and the transplanted kidney.

These reflect the constraints, both financial and physical to conservatively manage sirenomelia and reiterate the importance of antenatal diagnosis and voluntary termination of pregnancy especially in our resource limited settings like ours.

## Conclusion

Sirenomelia remains a rare but peculiar syndrome. Controversies on its etiopathogenesis persist. Its antenatal diagnosis is possible albeit difficult by ultrasound. The associated visceral anomalies are usually incompatible with life. However surviving sirenomelic foetuses have been described with costly conservative management and mediocre results. In the African context, these mermaid-like foetuses are described as ‘mammy-water babies’. This carries with it the connotation of sorcery and witchcraft with which no family wishes to be identified. Therefore antenatal diagnosis and termination of pregnancy is advisable. Knowledge of this rare syndrome is important to dissipate cultural myths whenever it occurs, and free the family from stigmatization.

## Consent

Written informed consent for publication of their clinical details and/or clinical images was obtained from the patient/parent/guardian/relative of the patient.
