# Supplementary material for: Sirenomelia in a Cameroonian woman: a case report and review of the literature
Source: F1000Res. 2012 Sep 6;1:6. Originally published 2012 Jul 26. [Version 2] doi: 10.12688/f1000research.1-6.v2 (PMC3917649; doi:10.12688/f1000research.1-6.v2)
Supplement: Peer review history

# Peer review: Referee response for version 2

**Published:** 2012-09-24

**Authors:** John Svigos

**Affiliations:** 1 Discipline of Obstetrics & Gynaecology, School of Paediatrics & Reproductive Health, Faculty of Health Sciences, University of Adelaide, Adelaide, Australia

The article reads much better now, and it represents the most comprehensive current review of sirenomelia in the available literature.

I have read this submission. I believe that I have an appropriate level of expertise to confirm that it is of an acceptable scientific standard.

# Peer review: Referee response for version 1

**Published:** 2012-07-27

**Authors:** John Svigos

**Affiliations:** 1 Discipline of Obstetrics & Gynaecology, School of Paediatrics & Reproductive Health, Faculty of Health Sciences, University of Adelaide, Adelaide, Australia

This is an interesting article with some quaint comments indicative of local community attitudes to bizarre abnormalities, which increase the appeal of the article.

The author(s) should consider being more specific about the actual incidence of the abnormality itself and the actual incidence associated with twins and diabetes. The author(s) should also review the level of detail under the ‘Ethiopathogenesis’ and ‘Classification’ subheadings, as I believe that these sections should be shortened/abbreviated if it is to fulfill the criteria of a ‘Case Report’ presentation.

I have read this submission. I believe that I have an appropriate level of expertise to confirm that it is of an acceptable scientific standard.

# Response

**Published:** 2012-09-12

**Authors:** Frederick Morfaw

**Affiliations:** Department of Obstetrics and Gynaecology, Faculty of Medicines and Biomedical Sciences, University of Yaoundé, Cameroon

Thank you for your very pertinent comments. With these taken on board we have now reviewed the work and made changes accordingly in a new version of the article.

# Peer review: Referee response for version 1

**Published:** 2012-07-26

**Authors:** Laxmi Baxi

**Affiliations:** 1 Department of Obstetrics and Gynecology, Columbia University Medical Center, New York, NY, USA

This is an interesting article. However there are a few things that need addressing:

1. There should be no judgmental statements.2. The author(s) should not confuse between gene and chromosomes.3. There should be more emphasis on scientific data and information.4. If the author(s) do need to address historical data, then it should be in full.

It is too bad there isn’t any pre- or post-birth imaging, however the photographs presented are interesting. There also seems to be a repetition of some facts, which need to be curtailed.

I have read this submission. I believe that I have an appropriate level of expertise to confirm that it is of an acceptable scientific standard.
